# Antibiotic use at planned central line removal in reducing neonatal post-catheter removal sepsis: a systematic review and meta-analysis

**DOI:** 10.3389/fped.2023.1324242

**Published:** 2024-01-08

**Authors:** Ruoyu Ji, Zhangyuting He, Jiawei Zhou, Shiyuan Fang, Lili Ge

**Affiliations:** ^1^ Department of Allergy, Peking Union Medical College Hospital, Chinese Academy of Medical Sciences & Peking Union Medical College, Beijing, China; ^2^ Department of Haematology, Peking Union Medical College Hospital, Chinese Academy of Medical Sciences & Peking Union Medical College, Beijing, China; ^3^ Department of Vascular Surgery, Peking Union Medical College Hospital, Chinese Academy of Medical Sciences & Peking Union Medical College, Beijing, China; ^4^ Department of Neurology, Peking Union Medical College Hospital, Chinese Academy of Medical Sciences & Peking Union Medical College, Beijing, China; ^5^ Department of Pediatrics (Neonatology), Yancheng Third People’s Hospital, Yancheng, China

**Keywords:** neonate, sepsis, critical care, central venous catheters, meta-analysis

## Abstract

**Background:**

Post-catheter removal sepsis (PCRS) is a notable complication of indwelling central venous catheters (CVCs) in neonates, which is postulated to be secondary to the disruption of biofilms formed along catheter tips up on CVCs removal. It remains controversial whether this could be prevented by antibiotic use upon CVCs removal. We aimed to evaluate the protective effect of antibiotic administration at the time of CVCs removal.

**Methods:**

We searched through PubMed, EMBASE, Cochrane databases and reference lists of review articles for studies comparing the use of antibiotics versus no use within 12 h of CVCs removal. Risk of bias was assessed using the modified Newcastle-Ottawa Scale and Cochrane risk-of-bias tool accordingly. Results of quantitative analyses were presented as mean differences (MD) or odds ratio (OR). Subgroup and univariate meta-regression analyses were performed to identify heterogeneity.

**Results:**

The review included 470 CVCs in the antibiotic group and 658 in the control group. Antibiotic use within 12 h of CVCs removal did not significantly reduce the incidence of PCRS (OR = 0.35, 95% CI: 0.08–1.53), but was associated with a lower incidence of post-catheter removal blood stream infection (OR = 0.31, 95% CI: 0.11–0.86). Dosage of vancomycin and world region were major sources of heterogeneity.

**Conclusion:**

Antibiotic administration upon CVCs removal does not significantly reduce the incidence of PCRS but offers less post-catheter removal blood stream infection. Whether this will be converted to better clinical outcomes lacks evidential support. Further randomized controlled studies with longer follow-up are needed.

**Summary:**

Results of our meta-analysis suggest that antibiotic use at planned central line removal removal does not significantly reduce the incidence of PCRS but offers less blood stream infection, which might contribute to future management of central lines in neonates.

**Systematic Review Registration:**

https://www.crd.york.ac.uk/, PROSPERO (CRD42022359677).

## Introduction

Central venous catheters (CVCs) are commonly used in the neonatal intensive care unit (NICU), contributing to better survival outcomes in critically ill newborn infants. Post-catheter removal sepsis (PCRS) is an important complication of indwelling CVCs with an incidence reported varying from 1.9% to 11.6% (1, 2). PCRS is predominantly caused by late-onset central line-associated blood stream infection (CLABSI) which is defined as a primary blood stream infection developing within 48 h after CVCs removal in the absence of other known infection sites (3). It is hypothesized that a biofilm forms along the inserted catheter, which is disrupted and washed into blood stream at the removal of CVCs, leading to bacteremia (2, 4). CLABSI is correlated with increased morbidity and mortality, additional antibiotic use and prolonged hospitalization (5, 6). Fortunately, implementation of central-line bundles and prophylactic systemic antibiotics use in the 72 h preceding PICC removal may help to reduce the incidence of late-onset CLABSIs (7, 8). However, the relatively long exposure to antibiotics during infancy is challenged by the selection of antibiotic-resistant organisms and gut microbiome dysbiosis, and thus prophylactic antibiotics use is not recommended (8, 9). Hence, concurrent antibiotics given at the time of CVCs removal might be an alternative strategy. Inconsistent results have been reported in several interventional or observational studies, which underscores the need to perform a systematic review and meta-analysis to quantitatively evaluate whether antibiotics administration at the time of CVCs removal prevents late-onset sepsis in neonates.

## Materials and methods

We performed the systematic review based on a protocol with the registration number CRD42022359677) and complied with the Preferred Reporting terms for Systematic Review and Meta-Analysis (PRISMA) statement (10). Reporting items were detailed in the PRISMA checklist (Supplementary Table S1).

The purpose of this review was to evaluate whether antibiotics administration within 12 h of planned CVCs removal can reduce the incidence of post-catheter removal sepsis in neonates.

### Literature search

We searched through PubMed, EMBASE and Cochrane databases. The search strategy in PubMed was: [central AND (catheter OR line)] AND (removal OR remove OR removing) AND (infection OR sepsis OR bacteremia) AND (infant OR neonate OR neonatus OR neonatal OR newborn) AND (antibiotic OR prevention OR prevent OR preventing OR prophylaxis OR prophylactic). The search strategy was adapted for EMBASE and Cochrane databases. We also searched references of review articles for relevant studies. The last search update was August 2023.

### Selection of studies

Studies were selected according to the PICOS (patients/participants, intervention, comparison, outcome, study type) approach. Inclusion criteria were:

Patients/participants: neonates aged ≤28 days admitted in NICU, undergoing planned removal of CVCs.

Intervention: antibiotics use within 12 h of planned CVCs removal.

Comparison: no antibiotics use within 12 h of planned CVCs removal.

Primary outcomes: PCRS which is defined as the appearance of clinical signs and symptoms of infection or the initiation of anti-infection therapy, with or without confirmatory blood markers or cultures within 72 h after catheter removal (11);

Secondary outcomes: (1) late-onset blood stream infection which is defined as clinical or laboratory signs of infection plus a positive blood culture or specific non-culture based microbiologic testing methods which is not related to the infection at another site (12); (2) CLABSI which is defined as clinical or laboratory signs of infection plus a positive blood culture developing within 48 h of CVCs removal in the absence of other known infection sites (3); (3) neonatal mortality.

Studies: retrospective or prospective human studies.

Exclusion criteria included: (1) noncomparative studies; (2) prophylactic antibiotics use for the duration of the CVCs; (3) therapeutic antibiotics use for known or suspected catheter-related bloodstream infections; (4) insufficient data for quantitative analyses; (5) grey literature lacking details or peer review. We set no restriction on language, publication type or date. Study selection was conducted by two researchers (RYJ and ZYTH) independently, with disagreements resolved through discussion with a senior investigator (LLG).

### Data extraction

We extracted the following data: (1) study information: publication (article title, authors, year, journal title), study design (patient inclusion and exclusion criteria, grouping, sample size of each) and bias control; (2) baseline characteristics: gestational age, sex, birth weight, races and country or region; (3) CVCs management: type, duration of insertion and indications for removal. (4) antibiotic use: type, dosage, frequency, start and end time. (5) outcomes: incidence of PCRS, late-onset blood stream infection and CLABSIs after catheter removal, neonatal mortality. Data extraction was conducted by two researchers (RYJ and ZYTH) independently, with disagreements resolved through discussion with a senior investigator (LLG).

### Risk of bias assessment

Risk of bias for randomized clinical trial (RCT) studies was assessed using the Cochrane risk-of-bias tool (13) based on seven domains: sequence generation, allocation concealment, blinding of participants and personnel, blinding of outcome assessment, incomplete outcome data, selective reporting and other bias (Supplementary Figure S1). For observational studies, risk of bias was assessed using a modified Newcastle-Ottawa Scale (NOS) (14) with the intention of best evaluating our phenomenon of interest (Supplementary Table S2). Assessment was performed based on three domains: selection, comparability and exposure, with a maximum score of 10. A total score of 5 or less, 6–7 and 8 or more was considered low, moderate and high quality, respectively. Risk of bias assessment was conducted by two researchers (RYJ and ZYTH) independently, with disagreements resolved through discussion with a senior investigator (LLG).

### Statistical analysis

Basic characteristics of enrolled studies were firstly tabulated. Variables reported by three or more studies were evaluated through quantitative analyses. For continuous data, the mean differences (MD) with 95% confidence intervals (CI) were calculated as the effect measurements. Data reported as the median with interquartile range were converted into the mean with standard deviation through a recommended formula (15). For binary data, the odds ratio (OR) and 95% CI were calculated as the effect measurements. Heterogeneity across studies were evaluated by Cochrane chi-square (*χ*^2^) and quantified with the *I*^2^ statistics (16). *I*^2^ values of 25, 50% and 75% represented low, moderate and high heterogeneity, respectively (17). For valuables with *I*^2^ values ≤25%, the fixed-effect model will be used, otherwise, we used the random-effect model for data synthesis. We performed the following subgroup analyses to explore sources of heterogeneity: gestational age, birth weight, duration of catheter insertion, world region, type of study design and type, dosage and frequency of antibiotics use. Univariate meta-regression analyses were further performed to identify heterogeneity sources across studies. Multivariate meta-regression analyses were not performed due to limited number of studies. Publication bias was not evaluated as no more than ten studies were enrolled (18). All analyses were performed using Review Manager 5.3.3 (Nordic Cochrane Centre, Copenhagen, Denmark) and *P* < 0.05 was considered statistically significant.

## Results

### Baseline characteristics

**Figure 1:**
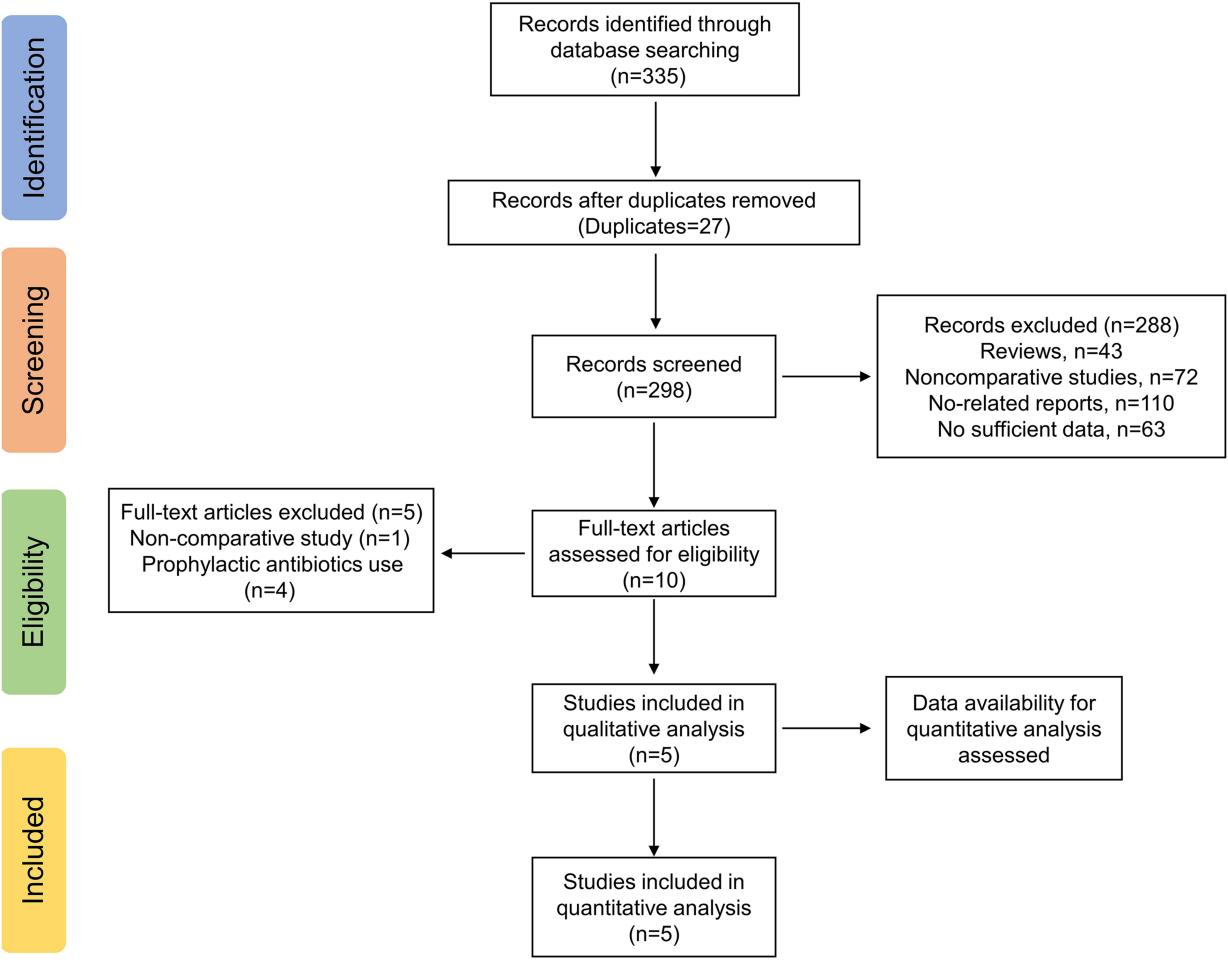
Study flow chart.

The electronic search yielded a total of 335 potentially relevant studies (Figure 1). All records were imported into the Endnote with 37 duplicates removed. After reading the titles and abstracts, 288 irrelevant studies were further eliminated. Among the remaining 10 studies, four studies regarding routine prophylactic or therapeutic antibiotics use (2, 19–21) and one non-comparative study (22) were excluded. Therefore, a total of five studies, including one RCT (23) and four retrospective studies (1, 24–26) were ultimately enrolled in the quantitative analyses (Table 1).

**Table 1:** Basic characteristics of included studies.

	Study design	Country/region	Antibiotic group					Control group						Definition for outcomes
			Number of CVCs	Male (%)	Gestational age (wks)	Birth weight (g)	Indwelling time (days)	Antibiotics use	Number of CVCs	Male (%)	Gestational age (wks)	Birth weight (g)	Indwelling time (days)	
Reynolds 2015	Retrospective	USA	48	NA	25.7 ± 2.1	743 ± 225	27.2 ± 11.4	Vancomycin (15 mg/kg) 2 h before catheter removal OR others within 12 h before catheter removal	165	NA	27.1 ± 2.5	897 ± 197	23.4 ± 11.3	PCRS: clinical sepsis event as the performance of a sepsis workup (white blood cell count, differential, CRP, blood and/or CSF, and urine cultures) along with antibiotics given for more than 48 h initiated within 72 h of CVCs removal as indicated by the medical team.
Hemels 2011	RCT	Netherland	44	66	30.0 ± 8.4	1730 ± 1908	13.0 ± 19.2	Cefazolin (50 mg/kg) 1 h before and 12 h after catheter removal	44	46	30.0 ± 7.7	1407 ± 1326	9.3 ± 10.7	Post-catheter removal blood stream infection: clinical signs of infection accompanied by laboratory abnormalities and a positive blood culture within 48 h of CVCs removal.
Teibel 2020	Retrospective	USA	107	55	33.8 ± 4.3	2316 ± 930	12.0 ± 9.5	Vancomycin (15 mg/kg) plus cefazolin (25 mg/kg) 2 h before catheter removal	109	50	33.8 ± 4.1	2256 ± 920	12.4 ± 10.6	PCRS: by a sepsis workup including ≥2 of the following: complete blood count with differential, CRP, blood culture, urine culture, CSF culture, or antibiotics given for more than 48 h within 72 h of CVCs removal.
Tran 2021	Retrospective	USA	14	60	30.7 ± 9.0	1400 ± 1554	39.3 ± 10.6	Vancomycin (15 mg/kg) 1.5 h before catheter removal	12	60	31.0 ± 8.2	1426 ± 1471	37.7 ± 7.4	PCRS: the initiation of antibiotics within 72 h of CVC removal and the collection of at least one of the following: complete blood count and differential, CRP, or a specimen culture (blood, urine, tracheal aspirate, and/or CSF).
Yan 2021^a^	Retrospective	Taiwan	257	NA	NA	NA	NA	Vancomycin (10 mg/kg) 2 h before catheter removal OR others within 12 h before catheter removal	328	NA	NA	NA	NA	Clinical sepsis: clinical symptoms of systemic illness, with initiation of antibiotics without a positive blood culture. A workup for sepsis includes complete blood count with differential, blood, CSF, and urine cultures; CRP measurement performed within 72 h of CVCs removal; and administration of antibiotics for at least 48 h.

^a^ Basic characteristics of this study were not obtained due to different groupings between this study and our meta-analysis.

RCT, randomized clinical trial; CVCs, central venous catheters; wks, weeks; hrs, hours; PCRS, post-catheter removal sepsis; CRP, C-reactive protein; CSF, cerebral spinal fluid; NA, not applicable.

All enrolled studies were conducted in the health setting of NICU. Altogether, 470 central lines in the antibiotic group and 658 central lines in the control group based on 1,054 neonates were included. The commonly used antibiotic regimen was a single dose of vancomycin (10 or 15 mg/kg) given at 2 h prior to CVCs removal. Other regimens included one dose of vancomycin plus cefazolin or two doses of cefazolin. There were no statistically significant differences between two groups in basic demographic characteristics including gestational age (Mean difference (MD) = −0.75 weeks, 95% confidence intervals (CI): −1.72 to 0.22, *P* = 0.13), male proportion (OR = 1.42, 95% CI: 0.91–2.22, *P* = 0.12) and birth weight (MD = −63.8 g, 95% CI: −232.6 to 105.0, *P* = 0.46). Also, the length of CVCs indwelling is comparable between two groups (MD = 1.61 days, 95% CI: −0.78 to 4.01, *P* = 0.19) (Table 2).

**Table 2:** Comparison of clinical characteristics.

Variables	Antibiotic vs. Control^a^	*I*^2^(%)	*P*
Gestational age MD (95% CI), weeks	−0.75 [−1.72, 0.22]	36	0.13
Male proportion OR (95% CI)	1.42 [0.91, 2.22]	0	0.12
Birth weight MD (95% CI), g	−63.8 [−232.6, 105.0]	32	0.46
CVCs indwelling time MD (95% CI), days	1.61 [−0.78, 4.01]	22	0.19

MD, mean differences; OR, odds ratio; CI, confidence interval.

^a^ A positive MD or OR favors antibiotic group.

### Incidence of PCRS

**Figure 2:**
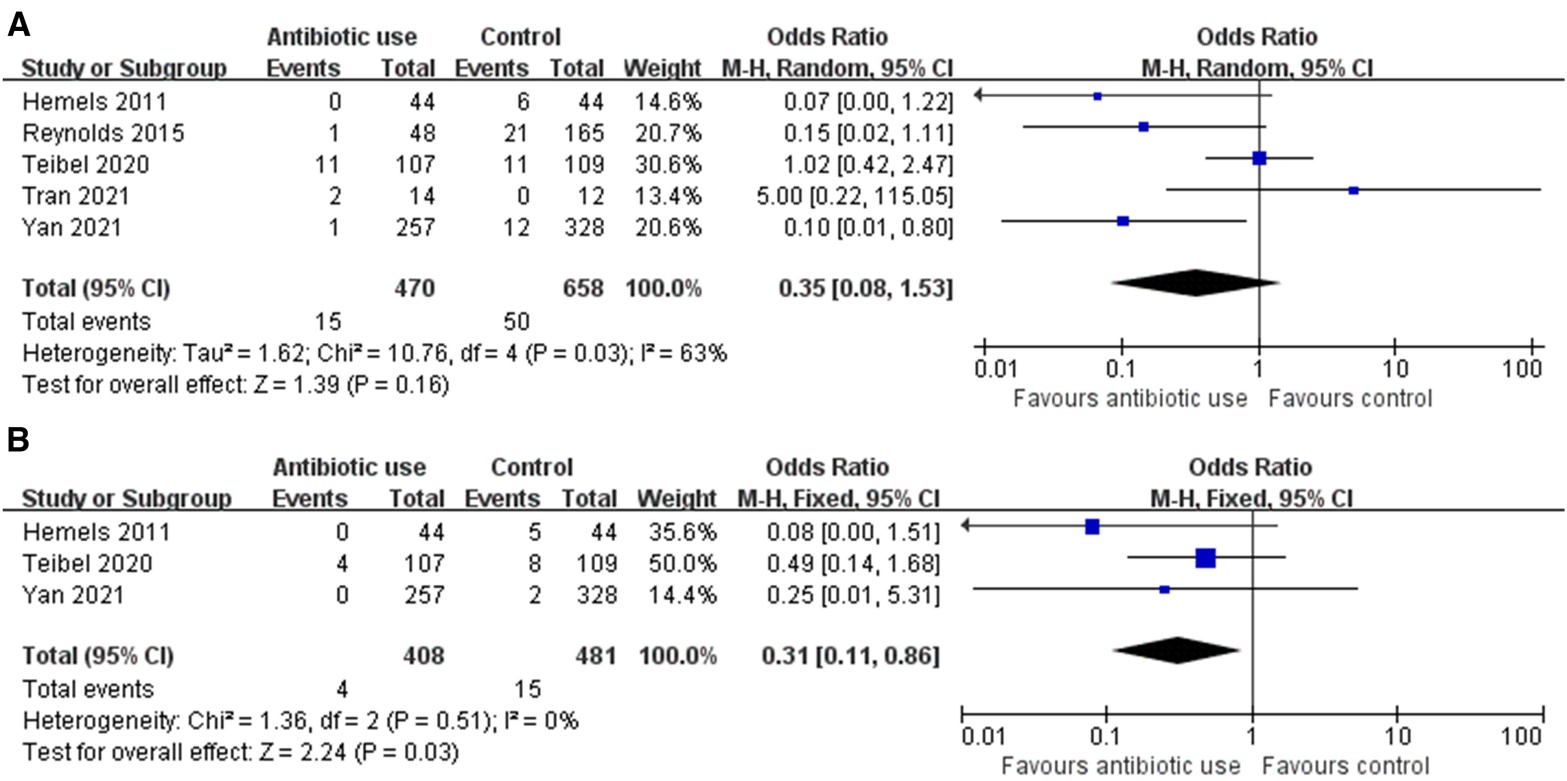
(**A**) Forrest plot of incidence of post-catheter removal sepsis (PCRS) for antibiotic use versus control. (**B**) Forrest plot of incidence of post-catheter removal blood stream infection for antibiotic use versus control.

In total, 15 of 470 (3.2%) and 50 of 658 (7.6%) CVCs removal episodes had PCRS in the antibiotic group and control group, respectively. The random-effects meta-analysis demonstrated that antibiotics given within 12 h of CVCs removal non-significantly reduced the incidence of PCRS (OR = 0.35, 95% CI: 0.08–1.53, *P* = 0.16, *I*^2^ = 63%) (Figure 2A).

Results of subgroup analyses and univariate meta-regression were detailed in Table 3. No significant subgroup difference was tested. Regarding antibiotic regimens, the vancomycin subgroup (OR = 0.63, 95% CI: 0.02–24.37) and the cefazolin subgroup (OR = 0.07, 95% CI: 0.00–1.22) showed non-significant protective effect, while the combination subgroup suggested a near equal incidence of PCRS (OR = 1.02, 95% CI: 0.42–2.47) with and without antibiotics use. In addition, the single-dose subgroup (OR = 0.56, 95% CI: 0.15–2.15) and two-doses (OR = 0.07, 95% CI: 0.00–1.22) subgroups both demonstrated non-significant protective effect. Similar results were reached in subgroup analyses according to gestational age, birth weight, types of study design and length of CVCs indwelling. In the Asian region and low-dose (10 mg/kg) vancomycin subgroups, a single-dose of vancomycin (10 mg/kg) given at 2 h prior to CVCs removal significantly reduced the incidence of PCRS (OR = 0.12, 95% CI: 0.02–0.92), but such significant protective effect was not observed in the Western region subgroup (OR = 0.49, 95% CI: 0.10–2.47), and the high-dose (15 mg/kg) vancomycin subgroup (OR = 0.87, 95% CI: 0.18–4.12).

**Table 3:** Subgroup analyses and univariate meta-regression analyses of PCRS

Groups	Subgroups	Studies (*n*)	OR [95% CI]^a^	*I^2^* (%)	*I*^2^_sub_ (%)^b^	*P* ^c^
Mean gestational age	≥28 weeks	3	0.74 [0.11, 5.12]	56	22	0.26
	<28 weeks	1	0.15 [0.02, 1.11]	–		
Mean birth weight	≥1,500 g	2	0.36 [0.02, 5.50]	71	0	0.77
	<1,500 g	2	0.69 [0.02, 21.80]	71		
Types of antibiotics	Vancomycin	2	0.63 [0.02, 24.37]	74	36	0.21
	Cefazolin	1	1.02 [0.42, 2.47]	–		
	Vancomycin plus cefazolin	1	0.07 [0.00, 1.22]	–		
Frequency of antibiotic administration	One dose	4	0.56 [0.15, 2.15]	52	42	0.19
	Two doses	1	0.07 [0.00, 1.22]	–		
Dosage of vancomycin	Low dose (10 mg/kg)	1	0.12 [0.02, 0.92]	–	56	0.13
	High dose (15 mg/kg)	3	0.87 [0.18, 4.12]	40		
Length of catheter indwelling	Long length (≥20 days)	2	0.69 [0.02, 21.80]	71	0	0.77
	Short length (<20 days)	2	0.36 [0.02, 5.50]	71		
Study design	Observational	4	0.56 [0.15, 2.15]	52	42	0.19
	RCT	1	0.07 [0.00, 1.22]	–		
World region	Asian	1	0.12 [0.02, 0.92]	–	11	0.29
	Western	4	0.49 [0.10, 2.47]	49		

PCRS, post-catheter removal sepsis; OR, odds ratio; CI, confidence interval; RCT, randomized clinical trial.

^a^ A positive OR favors antibiotic group.

^b^ Heterogeneity across subgroups.

^c^ *P* value of univariate meta-regression analyses which test for subgroup differences.

### Incidence of post-catheter removal blood stream infection

In total, 4 of 408 (0.1%) and 15 of 481 (3.1%) CVCs removal episodes resulted in post-catheter removal blood stream infection, as was proven by blood culture, in the antibiotic group and control group, respectively. The fixed-effects meta-analysis demonstrated that antibiotics given within 12 h of CVCs removal significantly reduced the incidence of post-catheter removal blood stream infection (OR = 0.31, 95% CI: 0.11–0.86, *P* = 0.03, *I*^2^ = 0%) (Figure 2B). Subgroup analysis was not performed due to limited number of relevant studies.

### Risk of bias assessment

Risk of bias of retrospective studies were assessed by a modified NOS (Supplementary Table S2). The total score of the four studies (1, 24–26) was 7, 8, 9, 7, respectively, indicating a moderate to low risk of bias. The risk of bias of the RCT (23) was assessed by using the Cochrane risk-of-bias tool, which was detailed in the Supplementary Table S1. This study is an open RCT with no detailed randomization and allocation procedures reported. Also, the actual enrolled number of patients was fewer than the planned value. Therefore, we considered this RCT to be at high risk of bias.

### Publication bias

Publication bias was not evaluated because of a lack of test power when ten or fewer studies are enrolled (18).

## Discussion

Conflicting evidence surrounds the use of antibiotic at the time of CVCs removal to prevent late-onset sepsis. In this meta-analysis, we quantitatively evaluated the preventive effect of antibiotic administration within 12 h of planned CVCs removal on late-onset sepsis based on five studies with a total of 1,128 central lines. Results demonstrated that antibiotic use upon CVCs removal did not significantly alter PCRS rates (OR = 0.35, 95% CI: 0.08–1.53), but was correlated with a lower incidence of post-catheter removal blood stream infection (OR = 0.31, 95% CI: 0.11–0.86).

Our results suggested a non-significant protective effect of antibiotic use at the time of CVCs removal in reducing rates of PCRS, with a pooled rate of 3.2%. Similar results were also found in most subgroup analyses. However, the evidence is still inadequate to examine this clinical issue as prospective, high-quality studies regarding this issue are largely insufficient. More attention has been devoted to prophylactic antibiotic use where antibiotics were given during the whole period of CVCs insertion or within 72 h prior to CVCs removal. A large retrospective study demonstrated a protective effect (OR = 0.26, *P* < 0.001) of prophylactic antibiotics in preventing culture-negative sepsis. In the intervention group, PCRS was found in 17 of the 322 (5.3%) central lines that were free from infection before removal (27). Inconsistently in an earlier RCT, infants were randomly assigned to receive amoxicillin prophylaxis or no antibiotic prior to CVCs removal (21). PCRS was found in 3 of 75 (4.0%) lines and 8 of 73 (11.0%) lines in two groups (*P* = 0.107), indicating non-significant benefit brought by routine antibiotic prophylaxis. A Cochrane meta-analysis enrolling three RCTs further affirmed the effect of prophylactic antibiotics in reducing rates of PCRS (RR = 0.40, 95% CI: 0.20–0.78), with a pooled PCRS rate of 8.8% (8). Though the absolute incidence of PCRS was comparable between antibiotic prophylaxis and antibiotics on CVCs removal, the superiority of one or another could not be determined due to a lack of comparative studies. However, there is no doubt that a single or two doses of antibiotics on CVCs removal could help to avoid antimicrobial resistance and microbiome dysbiosis brought by long-term antibiotic prophylaxis in neonates (9, 28).

Although the antibiotic use upon CVCs removal did not exhibit a significant protective effect against PCRS, it was correlated a lower risk of post-catheter removal blood stream infection (culture-positive sepsis), as suggested by our analysis. The specific mechanisms underlying this is unclear. It might be explained that the infusion of antibiotic disrupts the catheter biofilm formed along the catheter tip, decreasing the load of bacteria showered into blood stream upon catheter removal and therefore preventing culture-positive sepsis (27). However, a culture-negative sepsis could still be caused by the inflammatory response to unculturable bacteremia, especially by gram-negative bacteria which is largely non-susceptible to vancomycin. Though correlated with less blood stream infection, whether antibiotic use on CVCs removal will contribute to better clinical outcomes was not identified in our systematic review due to insufficient data reported. A recent large-scale meta-analysis indicated that despite similar mortality rate of sepsis shared by culture-positive and culture negative sepsis, patients with culture-positive sepsis had significantly longer hospitalization and mechanical ventilation duration (29). A retrospective study based on the pediatric setting reported a significantly lower mortality rate and organ-dysfunction in the culture-negative group (30). We thus speculate that antibiotic use on CVCs removal may bring clinical benefits by reducing the rate of culture-positive sepsis, which should be further examined with more relevant data reported.

To the best of our knowledge, this meta-analysis provides the most updated assessments of current evidence regarding the use of antibiotics at the time of CVCs removal in reducing late-onset sepsis. Despite this, several limitations exist. Due to the small number of published studies, we include both RCT and retrospective observational studies in our analyses, which might limit the quality of generated evidence. Even though the subgroup analysis based on types of study design did not detect significant interstudy heterogeneity, our results should still be interpreted with caution as the robustness and convincingness of subgroup analysis could be weakened by small number of included studies. The neonatal sepsis lacks a consensus definition, and its definition varies among enrolled studies. The updated regional practice manual and recommendations define CLABSI as a laboratory confirmed bloodstream infection where an eligible organism is identified, and an eligible central line (in place for over 48 h) is present, and further categorized the CLABSI into various types (12, 31). Once a global consensus definition is established, our outcome definition and study selection should be modified accordingly, and a re-analysis should be performed. Several critical clinical outcomes such as mortality rate, subsequent antibiotics, and other treatments for PCRS, length of stay as well as long-term outcomes are unable to evaluate due to insufficient data reported. Also, there is a moderate to high interstudy heterogeneity for the primary outcome, even though sources of heterogeneity were partly identified by subgroup analyses. Therefore, with continuous publication of articles, the update of the meta-analysis is still warranted to improve the above deficiencies.

## Conclusions

In conclusion, results of our review suggests that antibiotic administration in neonates within 12 h of planned CVCs removal does not significantly reduce the incidence of PCRS but offers less post-catheter removal blood stream infection. However, whether this will be converted to clinical benefits lacks evidential support. These findings should be interpreted with caution due to limitations stated above. The update of meta-analysis is warranted with more randomized designed studies having a longer follow-up performed.

## Data Availability

The original contributions presented in the study are included in the article/Supplementary Material, further inquiries can be directed to the corresponding author.
