# Supplementary material for: Antibiotic use at planned central line removal in reducing neonatal post-catheter removal sepsis: a systematic review and meta-analysis
Source: Front Pediatr. 2024 Jan 8;11:1324242. doi: 10.3389/fped.2023.1324242 (PMC10800366; doi:10.3389/fped.2023.1324242)
Supplement: Supplementary file 2 [file Table2.pdf]

Supplementary table 2 Risk of bias assessment for enrolled retrospective studies by the modified Newcastle-Ottawa Scale[#]

Studies	Selection				Comparability		Exposure/Outcomes				Total score	Quality
	Item 1	Item 2	Item 3	Item 4	Item 1	Item 2	Item 1	Item 2	Item 3	Item 4		
Reynold 2015	1	1	1	0	0	1	0	0	1	1	7	Moderate
Teibel 2020	1	1	1	0	1	1	0	0	1	1	8	High
Tran 2021	1	1	1	0	1	1	1	1	1	1	9	High
Yan 2021	1	0	1	0	1	1	0	0	1	1	7	Moderate

Items of the modified Newcastle-Ottawa Scale are listed below.

NEWCASTLE - OTTAWA QUALITY ASSESSMENT SCALE NON-RANDOMIZED STUDIES

Note: A study can be awarded a maximum of one star for each numbered item.

Selection

1) Representativeness of the exposed cohort

a) truly representative of the average neonates requiring central line insertions in the NICU✵

b) somewhat representative of the average neonates requiring central line insertions in the NICU

c) selected group of users, eg nurses, volunteers

d) no description of the derivation of the cohort

2) Selection of the non-exposed cohort

a) drawn from the same community as the exposed cohort ✵

b) drawn from a different source

c) no description of the derivation of the non-exposed cohort

3) Ascertainment of exposure

a) secure medical record ✵

b) structured interview

c) written self-report

d) no description

4) Demonstration that outcome of interest was not present at start of study

a) yes ✵

b) no

Comparability

Comparability of cohorts on the basis of the design or analysis

1) study controls for demographic characteristics including gestational age, sex, birth weight and ethnicity, *etc*✵

2) study controls for characteristics of central lines including types of central lines and length of catheterization, *etc*✵

Exposure/Outcomes

1) Ascertainment of exposure

a) secure record with details ✵

b) structured interview where blind to case/control status

c) interview not blinded to case/control status

d) written self-report or medical records

e) no description

2) <u>Clear definition and report of outcomes</u>

a) yes, based on consensus clinical criteria ✵

b) yes, based on self-definition

c) no description

3) <u>Was follow-up long enough for outcomes to occur</u>

a) yes (select an adequate follow up period for outcome of interest) ✵

b) no

4) <u>Adequacy of follow up of cohorts</u>

a) complete follow up - all subjects accounted for ✵

b) subjects lost to follow up unlikely to introduce bias

c) subjects lost to follow up likely to introduce bias and no description of those lost

d) no statement
