# Supplementary figures and images for: Antibiotic use at planned central line removal in reducing neonatal post-catheter removal sepsis: a systematic review and meta-analysis
Source: Front Pediatr. 2024 Jan 8;11:1324242. doi: 10.3389/fped.2023.1324242 (PMC10800366; doi:10.3389/fped.2023.1324242)

Supplementary Figure 1 Risk of bias graph of the included randomized controlled trial

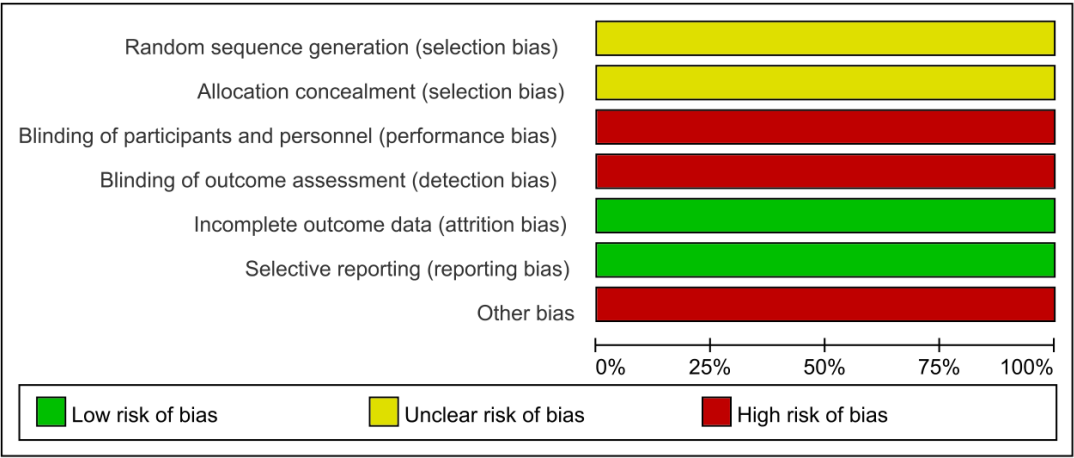

Supplement: Supplementary file 3 [file Image1.pdf]
